# FDG-PET as an independent biomarker for Alzheimer’s biological diagnosis: a longitudinal study

**DOI:** 10.1186/s13195-019-0512-1

**Published:** 2019-06-29

**Authors:** Ya-Nan Ou, Wei Xu, Jie-Qiong Li, Yu Guo, Mei Cui, Ke-Liang Chen, Yu-Yuan Huang, Qiang Dong, Lan Tan, Jin-Tai Yu, on behalf of Alzheimer’s Disease Neuroimaging Initiative

**Affiliations:** 1 Department of Neurology, Qingdao Municipal Hospital, Qingdao University, Qingdao, China; 2 Department of Neurology and Institute of Neurology, Huashan Hospital, Shanghai Medical College, Fudan University, 12th Wulumuqi Zhong Road, Shanghai, 200040 China

**Keywords:** ^18^F-Fluorodeoxyglucose positron emission tomography (FDG-PET), Alzheimer’s disease, ATN profile, Biomarker

## Abstract

**Background:**

Reduced ^18^F-fluorodeoxyglucose-positron emission tomography (FDG-PET) brain metabolism was recognized as a biomarker of neurodegeneration in the recently proposed ATN framework for Alzheimer’s disease (AD) biological definition. However, accumulating evidence suggested it is an independent biomarker, which is denoted as “F” in the very study.

**Methods:**

A total of 551 A+T+ individuals from the Alzheimer’s Disease Neuroimaging Initiative database were recruited and then further divided into four groups based on the biomarker positivity as 132 A+T+N−F−, 102 A+T+N−F+, 113 A+T+N+F−, and 204 A+T+N+F+. Frequency distributions of the groups were compared, as well as the clinical progression [measured by the longitudinal changes in cognition and brain structure, and mild cognitive impairment (MCI) to AD dementia conversion] between every pair of F+ and F− groups.

**Results:**

The prevalence of A+T+N+F+ profile was 66.24% in clinically diagnosed AD dementia patients; similarly, the majority of individuals with reduced FDG-PET were AD dementia subjects. Among the 551 individuals that included, 537 had at least one follow-up (varied from 1 to 8 years). Individuals in F+ groups performed worse and dropped faster in Mini-Mental State Examination scale and had faster shrinking middle temporal lobe than those in F− groups (all *p* < 0.05). Moreover, in MCI patients, reduced FDG-PET exerted 2.47 to 4.08-fold risk of AD dementia progression compared with those without significantly impaired FDG-PET (both *p* < 0.001).

**Conclusions:**

Based on the analyses, separating FDG-PET from “N” biomarker to build the ATN(F) system is necessary and well-founded. The analysis from this study could be a complement to the original ATN framework for AD’s biological definition.

**Electronic supplementary material:**

The online version of this article (10.1186/s13195-019-0512-1) contains supplementary material, which is available to authorized users.

## Background

Recently, the 2018 National Institute on Aging-Alzheimer’s Association (NIA-AA) proposed a research framework of a descriptive classification scheme for biomarkers used in Alzheimer’s disease (AD) research [1]. In this framework, “A” biomarkers refer to amyloidosis [abnormal tracer retention on amyloid positron emission tomography (PET) imaging and low β-amyloid (Aβ) concentration in the cerebrospinal fluid (CSF)]; “T” biomarkers, the value of increased CSF phosphorylated tau (P-tau) and cortical tau PET; and “N,” biomarkers of neurodegeneration or neural injury [higher CSF total tau (T-tau), diminished ^18^F-fluorodeoxyglucose (FDG)-PET and atrophic brain structures in magnetic resonance image (MRI)] [1]. An individual can be biologically diagnosed as AD when he/she present with both biomarker evidences of Aβ and pathological tau (A+T+). This framework treats AD as a continuum and introduces different pathologic biomarkers to weigh the diagnostic probability of the disease [2, 3].

FDG-PET is extensively and increasingly used to support the clinical diagnosis in the examination of patients with suspected neurodegenerative disorders, especially AD [4, 5]. It reflects both cumulative loss of neuropil, loss of synapse, and functional impairment of the neurons. Lower FDG-PET was regarded as a signal of neuronal hypometabolism due to neurodegeneration and was labeled as “N” biomarkers as the research framework defined. However, a recent study showed it reflects the consumption of glucose by astrocytes, rather than by neurons [6]. Moreover, there is a literature that has demonstrated that diminished FDG brain uptake by PET might be a biomarker tracking vascular, more precise, blood-brain barrier (BBB) transport, abnormality [7]. Based upon this hypothesis, an analysis was conducted in the Alzheimer’s Disease Neuroimaging Initiative (ADNI) prospective clinical cohort to explore the necessity and feasibility of making FDG-PET function as a separate biomarker, which is independent from “N” biomarker and labeled as “F” representing FDG hypometabolism in A+T+ individuals. This refinement enables an independent identification of non-specific neurodegenerative biomarkers to be independent, leading to a more precise understanding of the biological underpinnings of brain aging.

## Methods

### Participants

Data used in the preparation for this article were obtained from the ADNI database (http://adni.loni.usc.edu) [8, 9]. Individuals were included in our study if they underwent CSF Aβ (A), CSF P-tau (T), CSF T-tau (N), and FDG-PET (F) examinations at baseline. In an alternative analysis, we used adjusted hippocampal volume (HVa) to represent “N.” Individuals were classified into four groups: CN, MCI, which can be further divided into stable MCI (sMCI, MCI remained stable during at least 2 years follow-up) and progressive MCI (pMCI, who progressed from MCI to AD dementia during at least 2 years follow-up) and AD dementia. Patients with AD dementia fulfilled the National Institute of Neurological and Communicative Disorders and Stroke-Alzheimer’s Disease and Related Disorders Association criteria for probable AD, had Mini-Mental State Examination (MMSE) scores of 20 to 26, and had Clinical Dementia Rating (CDR) global scores of between 0.5 and 1.0. MCI patients had MMSE scores of 24 or higher, a CDR score of 0.5, objective memory loss tested by the delayed recall of the Wechsler Memory Scale logical memory II [> 1 standard deviation (SD) below the normative mean], preserved activities of daily living, and absence of dementia. Controls had MMSE scores of 24 or higher and a CDR global score of 0. Individuals with subjective memory complaints at baseline were not excluded from the analyses. Instead, they were included within the CN group.

### ATN(F) measurements

CSF samples were frozen on dry ice within 1 h after collection by lumbar puncture and shipped overnight on dry ice to the ADNI Biomarker Core laboratory. Aliquots (0.5 mL) were prepared from these samples and stored in barcode-labeled polypropylene vials at − 80 °C. Aβ-positive (A+) subjects were those who had CSF Aβ concentration levels ≤ 192 pg/ml [10]. Similarly, P-tau-positive (T+) was defined as a score above a cutoff point of 23 pg/ml [10]. T-tau-positive (T+) individuals were those who had CSF T-tau concentration levels ≥ 93 pg/ml [10]. We adjusted the hippocampal volume for total intracranial volume (TIV) using the following equation: HVa = Raw HV − *b* (eTIV − Mean eTIV), where *b* indicates the regression coefficient when HV is regressed against eTIV [11]. We defined HVa-positive (N+) and negative (N−) subjects based on a cutoff point of 6723 mm^3^ [11].

The FDG-PET images (via averaging counts of angular, temporal, and posterior cingulate regions) in this study were pre-processed using a series of steps to mitigate inter-scanner variability and obtain FDG-PET data with a uniform spatial resolution and intensity range for further analysis. Preprocessing steps included dynamic co-registration of images acquired in consecutive time frames, averaging, reorientation along the anterior-posterior commissure and filtering with a scanner-specific filter function to produce images of a uniform isotropic resolution of 8 mm full width at half maximum Gaussian kernel. A cutoff value equal to 1.21 was used to divide subjects into two groups: FDG-PET-negative subjects (F−) with level > 1.21 and FDG-PET-positive subjects (F+) with level ≤ 1.21 [10].

### Neuroimaging and cognition

Structural MRI was performed using a Siemens Trio 3.0 T scanner or Vision 1.5 T scanner (Siemens, Erlangen, Germany). Regional volume estimates were processed using Free-surfer software package version 4.3 and 5.1 image processing framework for the 1.5 and 3.0 T MRI images, respectively. We used middle temporal lobe (MTL) volume, entorhinal cortex (EC) thickness, and ventricular volume for measures of brain structure. General cognition was assessed by MMSE, Rey Auditory Verbal Learning Test (RAVLT)-immediate, and RAVLT-delayed recall scales.

### Statistical analysis

We adopted a two-step analysis in our report. Firstly, based on the cutoff thresholds of the four biomarkers, we dichotomized each biomarker category as either normal (−) or abnormal (+), which resulted in four different biomarker group combinations, including A+T+N−F−, A+T+N−F+, A+T+N+F−, and A+T+N+F+. The frequency distributions of the four clinical cognitive diagnosis and four ATN(F) profiles among different groups were summarized in the histogram. Tests of inter-group differences were performed using the chi-square analysis for frequencies or one-way analysis of variance and post hoc analysis for continuous measures. Categorical variables are presented as numbers (percents) and continuous variables as means ± SDs. Cognitive decline and brain atrophy over time were compared (A+T+N−F− vs A+T+N−F+ and A+T+N+F− vs A+T+N+F+ and A+T+N+F− vs A+T+N−F+) using linear mixed-effects models. Estimates with corresponding 95% confidence intervals (CIs) were obtained using a parametric bootstrap method in the arm package with 10,000 replicates. Analyses for the cognitive decline were adjusted for age, gender, *APOE* ε4, and years of education. Analyses for brain atrophy were adjusted for age, gender, *APOE* ε4, and total intracranial volume. Each clinical outcome measurement was log-transformed so that estimated change could be interpreted on an annual percentage scale. Unadjusted Kaplan-Meier (KM) analysis with the log-rank test to determine the progression from MCI to AD dementia was performed. Time-to-event was defined as the time from baseline MCI to AD dementia onset (with the end of study time or dropout as censor points). Additionally, the multivariate Cox proportional hazard model adjusted for baseline age, gender, educational level, and *APOE* ε4 status was conducted. Next, we compared clinical progression (measured by longitudinal cognitive/brain structure changes and MCI to AD dementia conversion) in the separate CSF T-tau (+), HVa (+), and FDG-PET (+) subgroups.

Statistical significance was defined as *p* < 0.05 for all analyses. Statistical analyses were performed using R (version 3.5.1) and IBM SPSS Statistics 23.

## Results

### Basic characteristics and inter-group comparisons

**Fig. 1:**
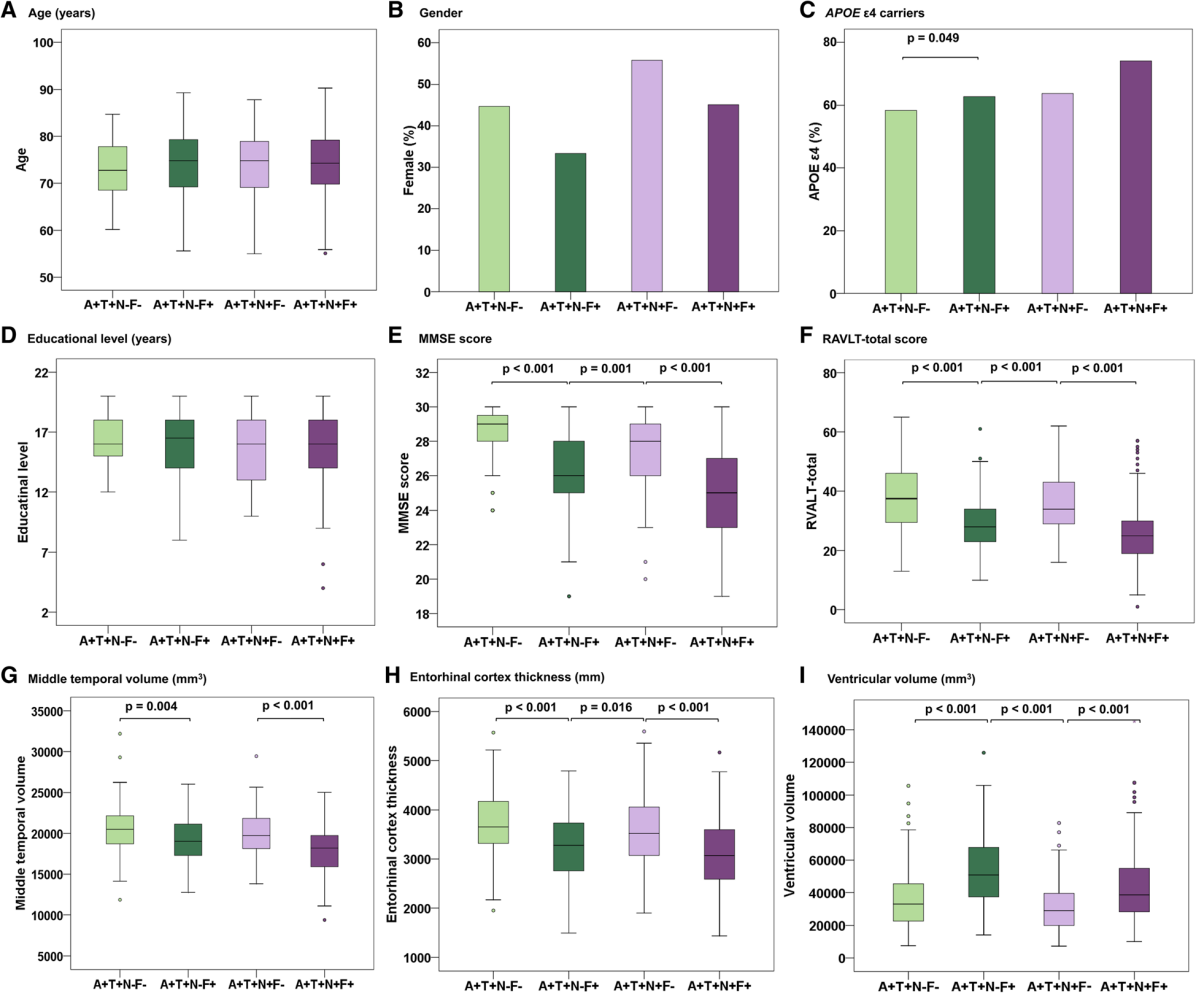
Plots of ATN(F) group characteristics. Box plots of continuous variables and bar charts summarized percentages of categorical variables from Table 1. As illustrated in Table 1, the four groups are arranged left-right hierarchically on the basis of A+T+, then the differences between F− vs F+ on the basis of N− and N+ were demonstrated. Significant *p* values of comparisons of every pair of F+ vs F− subgroups were depicted at the top of each figure. Abbreviations: MMSE, Mini-Mental State Examination; RAVLT-total, Rey Auditory Verbal Learning Test-immediate-total

Table 1 summarizes the basic demographic, clinical, psychometric, and CSF biomarker characteristics of our study population (*n* = 551, including 98 CN, 296 MCI, and 157 AD dementia). The study population had a female proportion of 38%, an age range from 55 to 90 years old at baseline (mean ± SD = 73.9 ± 7.2), education of 15.9 ± 2.8 years, and an *APOE* ε4 positive percentage of 67%. The difference in age between F+ and F− groups did not reach statistical significance (Table 1, Fig. 1a). F− groups had a relatively greater female proportion and this difference between F+ and F− groups did not reach significance (Table 1, Fig. 1b). The difference in *APOE* ε4 positive between N−F− group and N−F+ group reached statistical significance (*p* = 0.049; Fig. 1c) with a higher proportion of *APOE* ε4 carrier subjects in the F+ group. Overall, the A+T+N+F+ group had the highest proportion of *APOE* ε4 carrier subjects (74.02%; Table 1). Cognitive performances also differed to a great degree among groups (all *p* < 0.001; Table 1, Fig. 1e, f) with lower scores on MMSE and RAVLT-immediate in positive groups (lower scores represent worse cognitive function). Brain structures (MTL volume and EC thickness) significantly differed between groups, and they were smaller in F+ groups compared with F− groups (all *p* values < 0.05; Table 1, Fig. 1g, h) except for MTL volume in A+T+N−F+ and A+T+N+F− groups. However, the volume of ventricles in F+ groups was larger than that in F− groups (all *p* values < 0.001; Fig. 1i).

**Table 1:** Characteristics of participants by ATN(F) biomarker classification

Characteristics	A+T+N−F−	A+T+N−F+	A+T+N+F−	A+T+N+F+
*n*	132	102	113	204
Age (years)	73.21 ± 6.08	74.46 ± 7.38	74.09 ± 7.44	74.02 ± 7.59
Female (%)	59(45%)	34(33%)	63(56%)	92(45%)
Educational level (years)	16.31 ± 2.35	16.23 ± 2.89	15.57 ± 2.69	15.70 ± 2.94
*APOE* ε4 positive (%)	77(58%)	64(63%)	72(64%)	151(74%)
Cognitive score				
MMSE	28.21 ± 1.87	26.31 ± 2.56	27.62 ± 2.17	25.06 ± 2.89
RAVLT-immediate recall	37.98 ± 10.58	29.81 ± 10.22	35.29 ± 9.62	26.16 ± 9.87
RAVLT-delayed recall	3.48 ± 4.02	3.76 ± 4.13	3.51 ± 4.02	3.56 ± 4.05
Brain structure				
MTL volume (mm^3^)	20,603.68 ± 2852.81	19,230.37 ± 2999.68	20,078.17 ± 2789.14	17,863.85 ± 2891.62
EC thickness (mm)	3724.40 ± 635.16	3237.51 ± 715.711	3548.83 ± 738.51	3117.98 ± 724.23
Ventricular volume (mm^3^)	36,744.74 ± 18,700.67	55,002.57 ± 23,690.36	32,262.09 ± 18,079.57	42,955.53 ± 20,601.77
CSF Aβ (pg/ml)	142.71 ± 26.92	134.03 ± 25.24	136.06 ± 20.82	131.30 ± 22.54
CSF P-tau (pg/ml)	40.60 ± 15.28	41.69 ± 17.18	58.35 ± 22.56	62.99 ± 30.94
CSF T-tau (pg/ml)	67.05 ± 16.43	70.81 ± 15.45	142.66 ± 46.12	154.35 ± 55.96
FDG-PET	1.34 ± 0.09	1.09 ± 0.09	1.33 ± 0.09	1.07 ± 0.10

Categorical variables are reported as numbers and percentages; continuous variables are reported as means ± SDs

*Abbreviations*: *n* number, *SD* standard deviation, *MMSE* Mini-Mental State Examination, *RAVLT* Rey Auditory Verbal Learning Test, *MTL* middle temporal lobe, *EC* entorhinal cortex, *CSF* cerebrospinal fluid, *Aβ* β-amyloid, *P-tau* phosphorylated-tau, *T-tau* total-tau, *FDG-PET* ^18^F-fluorodeoxyglucose-positron emission tomography

The alternative analysis was also performed using HVa instead of CSF T-tau as the N measure (Additional file 1 and Additional file 2). The population size for this analysis (*n* = 493, including 90 CN, 260 MCI, and 143 AD dementia; 140 A+T+N−F−, 88 A+T+N−F+, 73 A+T+N+F−, and 192 A+T+N+F+) was smaller. There was high consistency between the two analyses using HVa and CSF T-tau separately. The two analyses had similarly significant differences in cognitive scores, EC thickness, and ventricular volume in both comparisons—N−F+ vs N−F− and N+F+ vs N+F− (all *p* values < 0.05). However, no significant differences were found between the N−F+ vs N+F− groups in all of the cognitive and brain structure measures except for EC thickness. This alternative analysis also found that only in the N+ category the volume of MTL in the F+ group was significantly lower than that in the F− group (*p* < 0.001).

### Frequency distributions of cognitive diagnosis and ATN(F) profiles

**Fig. 2:**
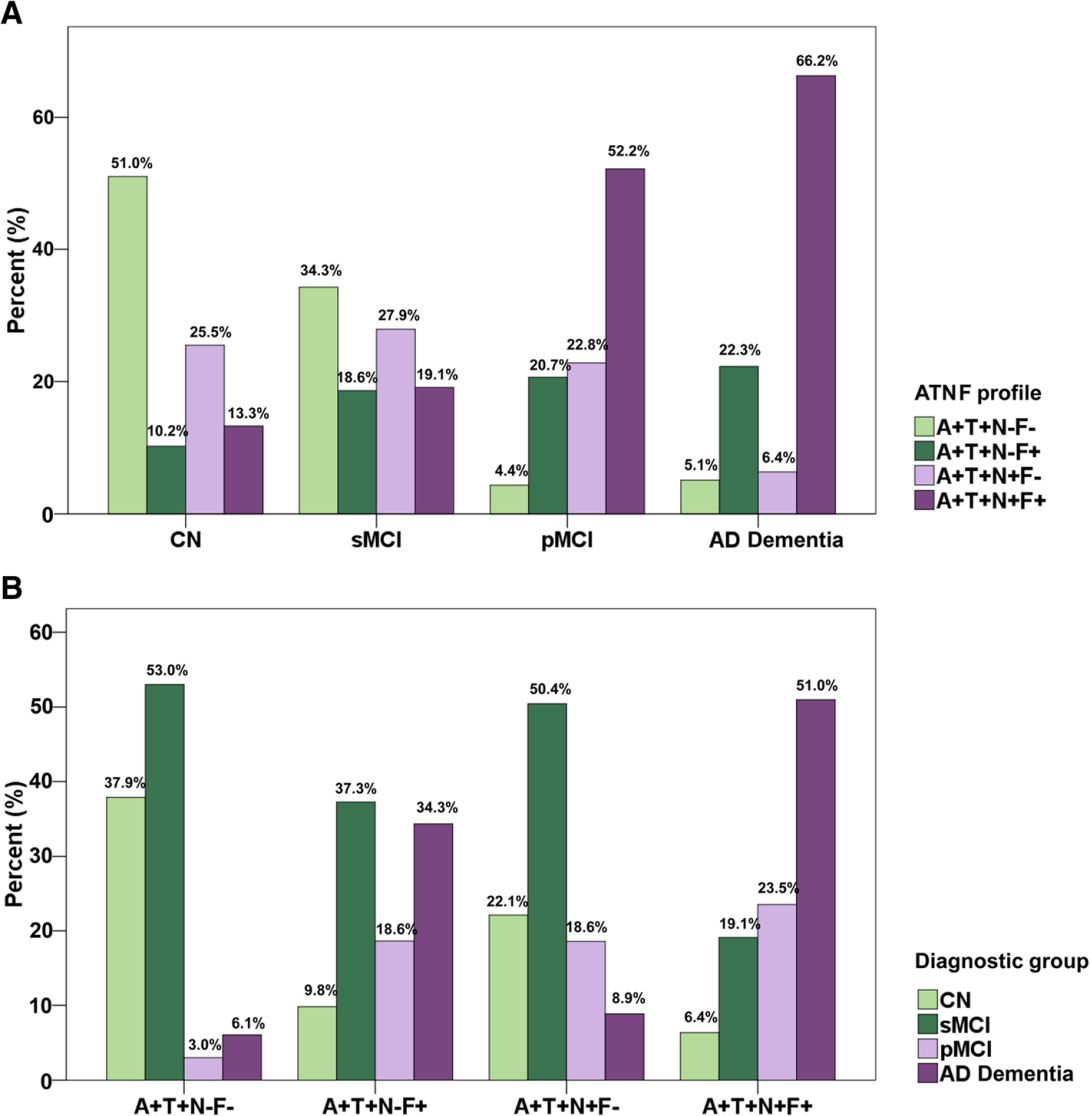
Frequency distributions of cognitive diagnosis and ATN(F) profiles among different groups. **a** The distributions of the four ATN(F) profiles in the population with different cognitive states. **b** Among the four ATN(F) groups, the various distributions of four cognitive states. Abbreviations: CN, cognitively normal; sMCI, stable mild cognitive impairment; pMCI, progressive mild cognitive impairment; AD, Alzheimer’s disease

The frequency distributions of clinical cognitive diagnosis among the four ATN(F) profiles and the frequency distributions of ATN(F) profiles among the four cognitive statuses are presented in Fig. 2. Abnormal FDG-PET (F+) accounted for 88.53% in AD dementia group (A+T+N−F+ accounted for 22.29% and A+T+N+F+ accounted for 66.24%; Fig. 2a). Similarly, in the pMCI group (those who progressed to AD dementia during at least 2 years follow-up), F+ accounted for 72.82% (A+T+N−F+ accounted for 20.65% and A+T+N+F+ accounted for 52.17%; Fig. 2a). From CN to AD dementia, the proportion of A+T+N+F+ was increasing (13.27% in CN, 19.12% in sMCI, 52.17% in pMCI, 66.24% in AD dementia), whereas the proportion of A+T+N−F− was decreasing (51.02% in CN, 34.31% in sMCI, 4.35% in pMCI, 5.10% in AD dementia) (Fig. 2a). Similarly, the F+ groups have a smaller proportion of CN (9.80% in N− group and 6.37% in N+ group) and a greater proportion of AD dementia (34.31% in N− group and 50.98% in N+ group) than the F− groups (Fig. 2b). From F− to F+, the proportion of AD dementia was increasing (6.06 to 34.31% in N− group and 8.85 to 50.98% in N+ group; Fig. 2b). Similar patterns of frequency distributions were depicted when HVa was used to define the N measure (see Additional file 3).

### Differences in longitudinal clinical outcomes

**Fig. 3:**
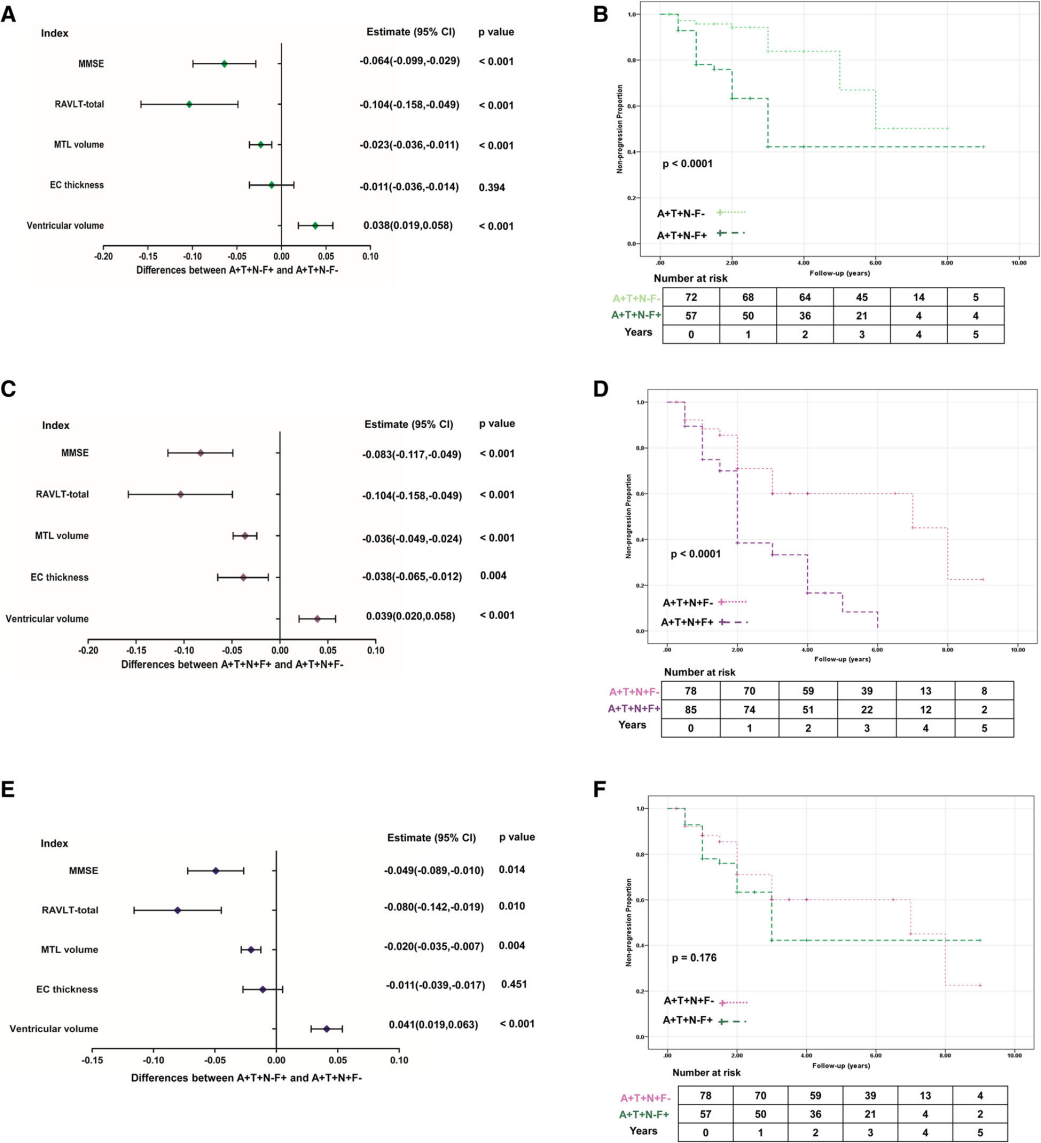
Clinical progression between every pair of F+ and F− groups. **a**, **c**, **e** The comparisons of longitudinal changes in cognitive performances and brain structure with A+T+N+F- vs A+T+N−F- revealed in **a**, A+T+N+F+ vs A+T+N+F- in **c**, and A+T+N-F+ vs A+T+N+F- in **e**. Differences between every pair of F+ and F− subgroups were demonstrated by estimates with 95% CIs and *p* values. Analyses of cognitive decline were adjusted for age, gender, *APOE* ε4, and years of education. Analyses of brain atrophy were adjusted for age, gender, *APOE* ε4, and total intracranial volume. **b**, **d**, **f** The Kaplan-Meier curves showing cumulative probability of MCI to AD dementia progression, which were arranged in accordance with the order mentioned above. The small crosses are censored data, and the number of subjects at risk is noted at the bottom of the plot. The unadjusted p values of log-rank test were depicted in the lower left. Abbreviations: MMSE, Mini-Mental State Examination; RAVLT-total, Rey Auditory Verbal Learning Test-immediate-total; MTL, middle temporal volume; EC, entorhinal cortex; MCI, mild cognitive impairment; AD Alzheimer’s disease

Of the 551 individuals, 537 subjects (95 CN, 292 MCI, and 150 AD dementia; 127 A+T+N−F−, 102 A+T+N−F+, 112 A+T+N+F−, and 196 A+T+N+F+) had at least one follow-up (varied from 1 to 8 years). The mean follow-up duration was 2.7 ± 1.6 years. Results of the linear mixed effect models for prediction of cognitive decline and brain atrophy over time were demonstrated in Fig. 3. Estimates represent differences in comparisons of A+T+N−F+ vs A+T+N−F- and A+T+N+F+ vs A+T+N+F- and A+T+N-F+ vs A+T+N+F-. The significant differences in rates of change in MMSE, RAVLT-immediate-total, MTL volume, and ventricular volume between F+ and F− in the A+T+N− group were presented in Fig. 3a (all *p* values < 0.001). Specifically, compared with F− group, F+ group had faster rates of reduction in MMSE [estimate = − 0.064, 95% confident interval (CI) = (− 0.099 to − 0.029), *p* < 0.001], RAVLT-immediate-total [estimate = − 0.104, 95% CI = (− 0.158 to − 0.049), *p* < 0.001] and MTL volume [estimate = − 0.023, 95% CI = (− 0.036 to − 0.011), *p* < 0.001], and a faster rate of expansion in ventricular volume [estimate = 0.038, 95% CI = (0.019 to 0.058), *p* < 0.001].

In A+T+N+ groups, the differences in rates of change in MMSE, RAVLT-immediate-total, MTL volume, EC thickness, and ventricular volume between F+ and F− groups were remarkable with all *p* values less than 0.005 (Fig. 3c). To be more specific, the F+ group dropped faster than the F− group in MMSE [estimate = − 0.083, 95% CI = (− 0.117 to − 0.049), *p* < 0.001], RAVLT-immediate-total [estimate = − 0.104, 95% CI = (− 0.158 to − 0.049), *p* < 0.001], MTL volume [estimate = − 0.036, 95% CI = (− 0.049 to − 0.024), *p* < 0.001] and EC thickness [estimate = − 0.038, 95% CI = (− 0.065 to − 0.012), *p* = 0.004], but increased faster in ventricular volume [estimate = 0.039, 95% CI = (0.020 to 0.058), *p* < 0.001].

We then compared the differences in rates of change in A+T+N-F+ and A+T+N+F- groups (Fig. 3e). Results demonstrated that the N−F+ group dropped faster than the N+F− group in MMSE [estimate = − 0.049, 95% CI = (− 0.089 to − 0.010), *p* = 0.014], RAVLT-immediate-total [estimate = − 0.080, 95% CI = (− 0.142 to − 0.019), *p* = 0.010], and MTL volume [estimate = − 0.020, 95% CI = (− 0.035 to − 0.007), *p* = 0.004], however increased faster in ventricular volume [estimate = 0.041, 95% CI = (0.019 to 0.063), *p* < 0.001].

When HVa was used to define the “N” biomarker, there were 217 subjects (35 CN, 136 MCI, and 46 AD dementia; 68 A+T+N−F−, 32 A+T+N−F+, 37 A+T+N+F−, and 80 A+T+N+F+) available for longitudinal analysis. The almost similar results were revealed (see Additional file 4A, C, E).

### Prediction of disease progression in MCI individuals

Of the 292 MCI patients (72 A+T+N−F−, 57 A+T+N−F+, 78 A+T+N+F−, and 85 A+T+N+F+), 129 progressed to AD dementia. In A+T+N− group, the KM curve indicated that the MCI patients who were classified in the F+ group had a higher risk of progression to AD dementia than those in the F− group (*p*_log-rank_ < 0.0001; Fig. 3b). Cox regression model adjusted for age, gender, educational level, and *APOE* ε4 status showed a robust result with hazard ratio (HR) = 4.08, 95% CI = (1.96–8.48). In A+T+N+ group, the result was similar. MCI patients with reduced FDG-PET uptake were more likely to progress to AD dementia with HR = 2.47, 95% CI = (1.55–3.93) than those without significantly impaired brain glucose metabolism (*p*_log-rank_ < 0.0001; Fig. 3d). Compared with N+F− group, N−F+ group showed no significantly increased risk of AD dementia conversion with HR = 1.53, 95% CI = (0.89–2.64), and *p*_log-rank_ = 0.176 (Fig. 3f).

When HVa was used to define the “N” biomarker, of the 136 MCI patients (45 A+T+N−F−, 22 A+T+N−F+, 27 A+T+N+F−, and 42 A+T+N+F+), 55 participants progressed to AD dementia. In A+T+N− group, MCI patients in the F+ group had much higher rates of progression to AD dementia than those in the F− group (*p*_log-rank_ = 0.018; see Additional file 4B). However, the result of multi-adjusted Cox regression was not statistically significant with HR = 2.40, 95% CI = (0.94–6.13). In A+T+N+ group, the result was similar with significant *p* value (*p*_log-rank_ = 0.019), adjusted HR of 1.94, and 95% CI of 0.88–4.27 (see Additional file 4D). Compared with the N+F− group, the N−F+ group showed no increased risk of AD dementia conversion with *p*_log-rank_ = 0.320 and HR = 1.38, 95% CI = (0.47–4.07) (see Additional file 4F).

### Clinical relevance and potential differences of using CSF T-tau vs HVa vs FDG-PET hypometabolism as “N”

Reduced brain FDG-PET metabolism showed strong relevance with cognitive decline measured by MMSE [estimate = 0.151, 95% CI = (0.114 to 0.188), *p* < 0.001], MTL atrophy [estimate = 0.035, 95% CI = (0.017 to 0.053), *p* = 0.001], and ventricular volume expansion [estimate = − 0.053, 95% CI = (− 0.079 to − 0.027), *p* < 0.001]. In comparison, atrophic HV seen on MRI was correlated with a drop in MMSE score [estimate = 0.038, 95% CI = (0.008 to 0.068), *p* = 0.014] and EC atrophy [estimate = 0.046, 95% CI = (0.013 to 0.079), *p* = 0.007]. Elevated CSF T-tau was related to MTL atrophy [estimate = − 0.006, 95% CI = (− 0.011 to − 0.001), *p* = 0.019] and large ventricular volume [estimate = 0.015, 95% CI = (0.009 to 0.023), *p* < 0.001] (see Additional file 5 A).

A total of 279 MCI individuals were included in the KM analysis. In detail, 148 patients were T-tau (+), 69 were HVa (+), and 62 were FDG-PET (+), of which 91/35/36 progressed to AD dementia eventually. We failed to detect the significant differences of clinical progression among these three groups (see Additional file 5B).

## Discussion

The primary finding is that cognitive decline and brain atrophy in A+T+ individuals with reduced FDG-PET brain metabolism are much faster than that in the individuals without significantly impaired FDG-PET uptake. Moreover, MCI patients with diminished FDG-PET have much higher rates of progression to AD dementia. Therefore, it is recommended to treat FDG-PET as an independent biomarker for the ATN(F) framework system.

Individuals with abnormal FDG-PET accounted for 88.53% of all the participants in AD dementia group and accounted for 72.82% in the pMCI group (those who progressed to AD dementia during at least 2 years follow-up). We also discovered that the proportion of A+T+N+F+ was increasing (13.27% in CN, 19.12% in sMCI, 52.17% in pMCI, 66.24% in AD dementia) and the proportion of A+T+N−F− was decreasing (51.02% in CN, 34.31% in sMCI, 4.35% in pMCI, 5.10% in AD dementia) from CN to AD dementia. The above results suggest that individuals with diminished glucose metabolism measured by PET are more likely to progress to AD or to be AD. Diminished glucose uptake in the hippocampus, parieto-temporal cortex, and/or posterior cingulate cortex has been repeatedly shown by FDG-PET in early AD [12] and/or MCI or no cognitive impairment before progression to AD dementia [13]. However, the sensitivity of FDG-PET index in our analysis only reaches about 72.82% in patients with pMCI, which is a rather low figure as compared to more sound volume of interest tools. More sensitive alternative choices tracking FDG-PET hypometabolism such as the Support Vector Machine model-based analysis need to be considered in the future studies [14, 15]. Similarly, reduced FDG-PET accounted for a relatively large proportion of AD dementia (34.31% in N− group and 50.98% in N+ group). From F− to F+, the proportion of AD dementia increased in both N− and N+ groups. These results were in line with the previous findings that AD subjects were more inclined to have reduced brain FDG uptake on PET [16–21].

Longitudinally, we used cognitive scales and MRI scans to investigate declines in brain function (cognitive function reductions) and structure (brain volume loss). MMSE scale, commonly believed to reflect the overall cognitive function and predict MCI to AD dementia progression [22], was chosen in our analysis. We also chose RAVLT to reflect psychological function in that word list learning is a predictor of conversion compared to other neuropsychological tests [23–26]. Consistent with our expectations and prior published results [27], our analysis discovered that individuals with lower FDG-PET metabolism had worse cognitive performances (scored lower at baseline and dropped faster over time on the two scales), hinting that diminished FDG on PET predicted or accelerated cognitive decline. Longitudinal FDG-PET findings have also suggested that reductions in hippocampal glucose uptake during normal aging can predict cognitive decline years in advance of clinical AD diagnosis [12]. The two scales are commonly used in evaluating the severity of cognitive impairment. The positive associations of reduced FDG-PET with these two scales could help us get a better understanding of the predictive value of FDG-PET.

Moreover, reduced FDG-PET was associated with reduced sizes of brain structures (smaller volumes at baseline and faster shrunk MTL volume and EC thickness longitudinally) and expanded ventricular volume. Cerebral atrophy typically starts from the MTL and limbic areas, spreads to parietal association areas, and finally progresses to frontal and primary cortices [28]. The pattern of hypometabolism in AD is that the posterior cingulate cortex is the first area affected, followed by the parieto-temporal areas and then by the frontal regions [29]. It was traditionally believed that cortical atrophy in AD showed a trajectory that markedly overlapped that of brain glucose metabolism [30]. However, recent studies support that diminished cerebral metabolic rate of glucose (CMRglu) measured by PET precedes cognitive decline and gray matter atrophy measured by MRI [31–36]. Among the three encephalic regions closely related to AD development in our analysis, the integrity of MTL is vital for memory function [37] and decreased MTL metabolism might be a specific marker of subclinical changes in cognition in preclinical AD [38]. Furthermore, reduced FDG uptake in EC is a known feature of AD [32, 39]. De Leon et al. showed that in healthy elderly subjects, reduction in cerebral glucose metabolism in EC predicted memory decline and temporal cortex metabolic reductions [21]. Our study suggests that lower FDG-PET predicts or accelerates brain atrophy, which is in line with the previous findings [16–21]. Brain glucose metabolism detected by PET is a potentially promising predictor for cerebral matter loss in AD, which could be employed as a valuable tool in clinical settings or scientific researches.

Individuals with MCI are a target population for evaluating very early treatment interventions for AD since they are at an intermediate stage between normal cognitive function and AD dementia, and are at higher risk of cognitive decline than healthy older individuals [13]. Several previous FDG-PET studies on MCI have focused on patients with clear-cut memory deficits (i.e., amnesic MCI) that were at high risk of developing AD dementia [40]. Our KM analysis indicates that the MCI patients who are labeled as A+T+F+ (with N+ or N−) have much higher rates of progression to AD dementia than A+T+F−. This might give us a hint that reduced FDG-PET may increase the probability of progression from MCI to AD dementia, which was exactly concordant with previous reports which showed that FDG-PET was a very sensitive measure for predicting conversion to AD dementia in patients with MCI [13], [41, 42].

In this study, CSF T-tau and adjusted HV were used as the “N” biomarkers, from which similar results were obtained. According to analyses, separating FDG-PET from the previous “N” biomarkers and further building the ATN(F) framework is necessary and well-founded. In the recently proposed biological definition of AD, the “N” represents a non-specific biomarker of neurodegeneration or neural injury, including CSF T-tau, atrophic brain structures seen on MRI, and FDG hypometabolism [1]. These three biomarkers may have discordances. CSF T-Tau probably indicates the intensity of neuronal injury at a given time point, MRI reflects cumulative loss and shrinkage of the neuropil, while FDG-PET conventionally reflects both cumulative loss of neuropil, loss of synapse and functional impairment of the neurons. In this study, the clinical relevance and potential differences of the above three “N” biomarkers were compared, and it is found that brain FDG-PET hypometabolism showed significant correlations with cognitive decline and brain atrophy. Additionally, all of the three biomarkers demonstrated a high correlation with increased risk of AD dementia conversion; however, the differences are not statistically significant. All these results suggest that FDG-PET may more indicative than the other two “N” biomarkers.

The generated results support the hypothesis that diminished FDG-PET should be re-considered as an independent biomarker. Actually, FDG hypometabolism is a summation of multiple biological processes, but not just neuronal hypometabolism and neurodegeneration. A recent review deems that the diminished uptake of FDG in the AD brain might point to a vascular deficit, specifically, impaired blood-brain barrier (BBB) function. Mechanically speaking, brain uptake of FDG depends on the glucose transporter 1 (GLUT1) [43, 44], which is only expressed on the endothelium of BBB. So, FDG hypometabolism might be a manifestation of BBB breakdown, which is regarded as an early biomarker of cognitive dysfunction that independent of Aβ and tau [45]. Moreover, accumulating publications reported that GLUT1 levels are substantially reduced in AD brain microvessels [46–49]. There is also a recent study indicated that it should be the astrocytes, instead of neurons, to be recognized as the contributor to the FDG signal [6]. In a word, the mechanism of action of FDG-PET is indeed much more complex than existing understanding, and FDG-PET hypometabolism should not be obtrusively classified as a “N” biomarker. This re-classification proposed in the present study may be of great significance to the diagnosis and treatment of patients with AD because it highlights the potential of FDG uptake to identify the therapeutic window before irreversible neurodegenerative attacks.

This study had limitations. Firstly, dichotomizing each biomarker might conceal an underlying continuum. With four different biomarkers employed, the classification error rate increased compared to a situation where only a single biomarker is used. Secondly, the CSF samples available for longitudinal analysis was insufficient, especially when the population was divided into four groups, which may limit the statistical power to detect longitudinal changes. Lastly, the sensitivity of FDG-PET index in patients with MCI due to AD was rather low, so automatic tools for the detection of Alzheimer-related hypometabolic pattern with high reliability and generalizability are in command.

## Conclusions

Taken together, the findings, as set forth, from the very study, suggest that A+T+ individuals with reduced brain FDG uptake on PET have worse cognition, accelerated brain atrophy and an increased likelihood of progression from MCI to AD dementia. It is concluded and recommended to regard FDG-PET as a separate biomarker, which is independent from the “N” biomarkers, in a ATN(F) framework system. The work could be a complement to completing the ATN descriptive framework.

## Additional files

Additional file 1: **Table S1.** Characteristics of participants by ATN(F) biomarker classification. (DOCX 20 kb)

Additional file 2: Plots of ATN(F) group characteristics. (DOCX 226 kb)

Additional file 3: Frequency distributions of cognitive diagnosis and ATN(F) profiles among different groups. (DOCX 276 kb)

Additional file 4: Clinical progression between every pair of F+ and F− groups. (DOCX 265 kb)

Additional file 5: Comparison of clinical progression between CSF T-tau (+) vs HVa (+) vs FDG-PET (+). (DOCX 1321 kb)

## Data Availability

The dataset generated and analyzed in the current study is available from the corresponding author on reasonable request.

## Abbreviations

AD: Alzheimer’s disease
ADNI: Alzheimer’s disease Neuroimaging Initiative
Aβ: β-amyloid
BBB: Blood-brain barrier
CDR: Clinical Dementia Rating
CI: Confidence interval
CMRglu: Cerebral metabolic rate of glucose
CN: Cognitively normal
CSF: Cerebrospinal fluid
EC: Entorhinal cortex
GLUT1: Glucose transporter 1
HVa: Adjusted hippocampal volume
KM: Kaplan-Meier
MCI: Mild cognitive impairment
MMSE: Mini Mental State Examination
MRI: Magnetic resonance image
MTL: Middle temporal lobe
NIA-AA: National Institute on Aging-Alzheimer’s Association
PET: Positron emission tomography
pMCI: Progressive MCI
P-tau: Phosphorylated tau
RAVLT-total: Rey Auditory Verbal Learning Test-total
SD: Standard deviation
sMCI: Stable MCI
TIV: Total intracranial volume
T-tau: Total tau
