# Supplementary material for: FDG-PET as an independent biomarker for Alzheimer’s biological diagnosis: a longitudinal study
Source: Alzheimers Res Ther. 2019 Jun 29;11:57. doi: 10.1186/s13195-019-0512-1 (PMC6599313; doi:10.1186/s13195-019-0512-1)
Supplement: Supplementary file 1 — Table S1. Characteristics of participants by ATN(F) biomarker classification. (DOCX 20 kb) [file 13195_2019_512_MOESM1_ESM.docx]

**Additional file 1 Table S1 Characteristics of participants by ATN(F) biomarker classification**

| Characteristics | A+T+N-F- | A+T+N-F+ | A+T+N+F- | A+T+N+F+ |
| --- | --- | --- | --- | --- |
| n | 140 | 88 | 73 | 192 |
| Age (years) | 72.28±6.41 | 70.88±7.80 | 76.61±5.73 | 75.08±7.15 |
| Female (%) | 71(51%) | 30(34%) | 38(52%) | 83(43%) |
| Educational level (years) | 16.01±2.49 | 16.88±2.22 | 15.68±2.55 | 15.48±3.00 |
| *APOE* ε4 positive (%) | 80(57%) | 53(60%) | 45(62%) | 145(76%) |
| Cognitive score |  |  |  |  |
| MMSE | 28.44±1.57 | 27.05±2.65 | 27.18±2.32 | 24.77±2.78 |
| RAVLT-immediate recall | 39.03±9.64 | 33.35±12.46 | 33.10±9.43 | 25.20±8.09 |
| RAVLT-delayed recall | 5.49±3.84 | 3.79±3.92 | 2.69±3.23 | 1.15±1.94 |
| Brain structure |  |  |  |  |
| MTL volume (mm^3^) | 20769.45±2730.86 | 19900.46±2579.13 | 19168.79±2588.46 | 17471.06±2787.77 |
| EC thickness (mm) | 3895.30±576.17 | 3644.13±571.04 | 3159.99±610.10 | 2927.74±674.90 |
| Ventricular volume (mm^3^) | 31118.13±18169.10 | 39957.67±20741.33 | 40296.11±17308.72 | 49505.62±22504.46 |
| CSF Aβ (pg/ml) | 142.00±23.76 | 136.72±24.77 | 137.10±24.87 | 129.48±21.85 |
| CSF P-tau (pg/ml) | 50.12±22.75 | 56.63±29.86 | 46.66±16.02 | 57.36±30.06 |
| Adjusted HV (mm^3^) | 7602.03±639.53 | 7307.90±465.73 | 5947.57±554.05 | 5709.21±659.40 |
| FDG-PET | 1.35±0.09 | 1.10±0.11 | 1.31±0.09 | 1.07±0.10 |

Categorical variables are reported as numbers and percentages; continuous variables are reported as means ± SDs.

Abbreviations: n: number, SD: standard deviation, MMSE: Mini-Mental State Examination, RAVLT: Rey Auditory Verbal Learning Test, MTL: middle temporal lobe, EC: entorhinal cortex, CSF: cerebrospinal fluid, Aβ: β-Amyloid, P-tau: phosphorylated-tau, HV: hippocampal volume, FDG-PET: ^18^F-fluorodeoxyglucose-positron emission tomography.
