# Supplementary material for: FDG-PET as an independent biomarker for Alzheimer’s biological diagnosis: a longitudinal study
Source: Alzheimers Res Ther. 2019 Jun 29;11:57. doi: 10.1186/s13195-019-0512-1 (PMC6599313; doi:10.1186/s13195-019-0512-1)
Supplement: Supplementary file 2 — Plots of ATN(F) group characteristics. (DOCX 226 kb) [file 13195_2019_512_MOESM2_ESM.docx]

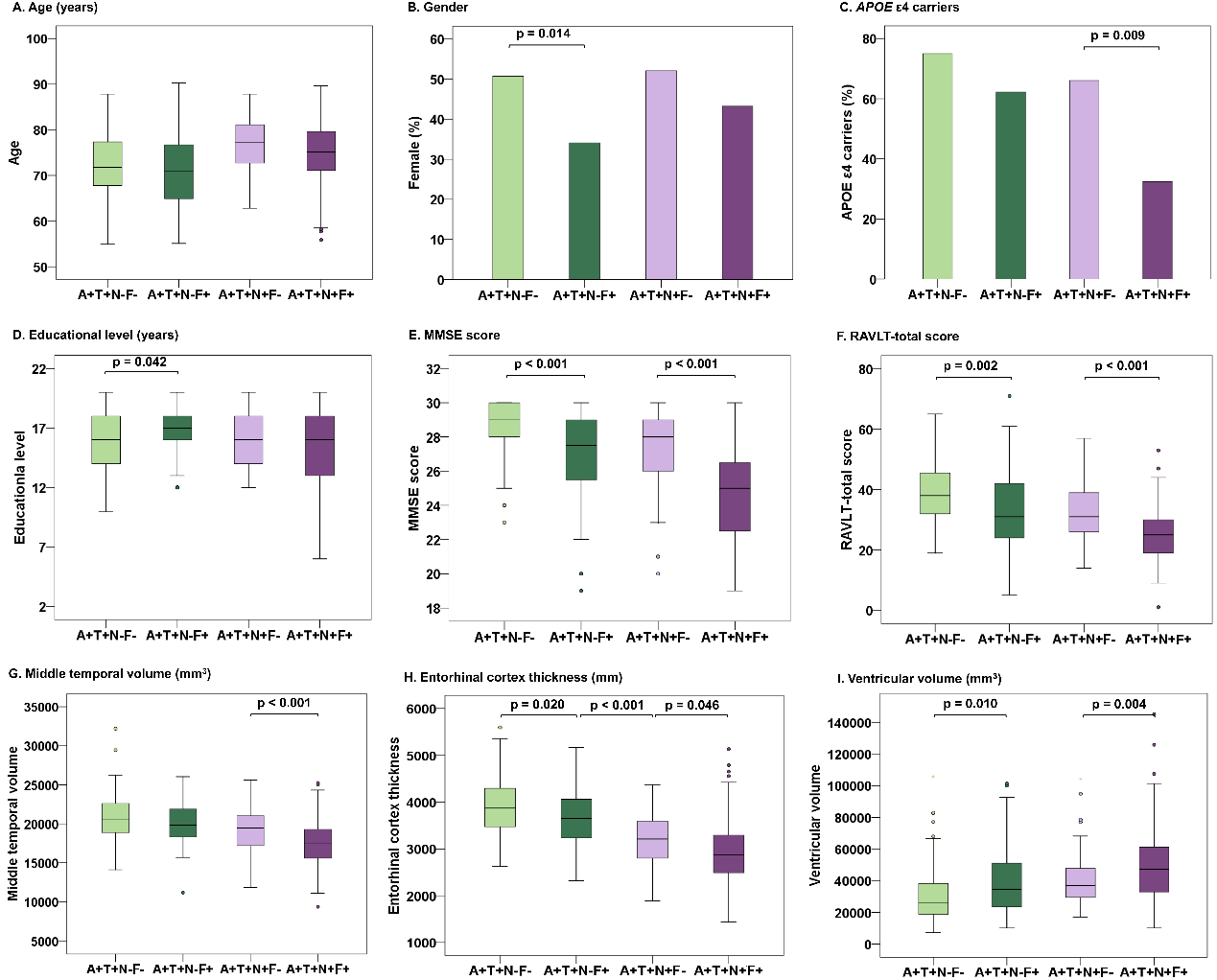


**Additional file 2 Plots of ATN(F) group characteristics**

Box plots of continuous variables and bar charts summarized percentages of categorical variables from Additional file 1. As illustrated in Additional file 1, the four groups are arranged left-right hierarchically on the basis of A+T+, then the differences between F- vs F+ on the basis of N- and N+ were demonstrated. Significant p values of comparisons of every pair of F+ vs F- subgroups were depicted at the top of each figure.

Abbreviations: MMSE: Mini-Mental State Examination, RAVLT: Rey Auditory Verbal Learning Test.
