# Supplementary material for: FDG-PET as an independent biomarker for Alzheimer’s biological diagnosis: a longitudinal study
Source: Alzheimers Res Ther. 2019 Jun 29;11:57. doi: 10.1186/s13195-019-0512-1 (PMC6599313; doi:10.1186/s13195-019-0512-1)
Supplement: Supplementary file 3 — Frequency distributions of cognitive diagnosis and ATN(F) profiles among different groups. (DOCX 276 kb) [file 13195_2019_512_MOESM3_ESM.docx]

**
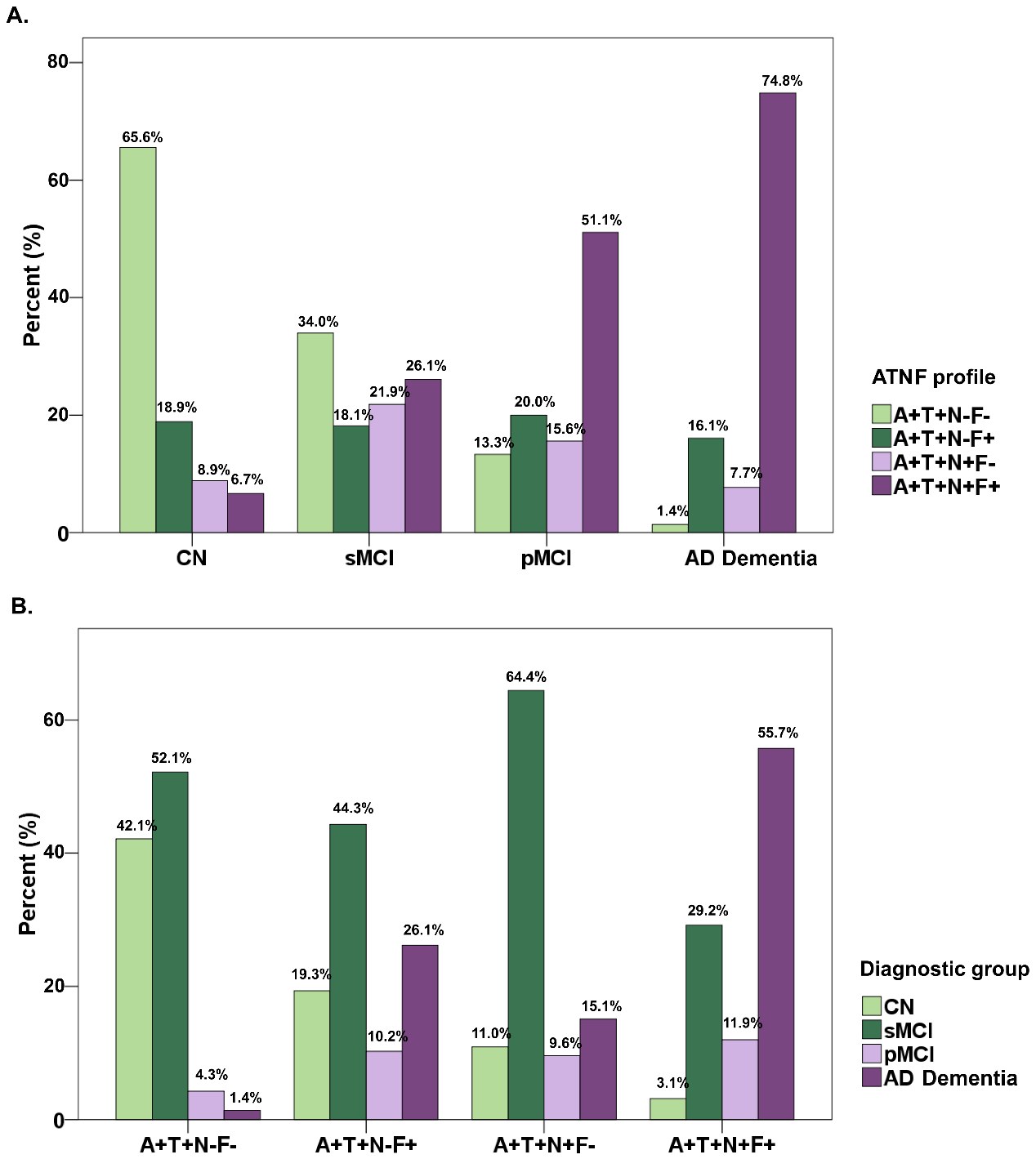
**

**Additional file 3 Frequency distributions of cognitive diagnosis and ATN(F) profiles among different groups**

Panel A shows the distributions of the four ATN(F) profiles in the population with different cognitive states. Panel B shows among the four ATN(F) groups, the various distributions of four cognitive states.

Abbreviations: CN: cognitively normal, sMCI: stable mild cognitive impairment, pMCI: progressive mild cognitive impairment, AD: Alzheimer’s disease.
