# Supplementary material for: FDG-PET as an independent biomarker for Alzheimer’s biological diagnosis: a longitudinal study
Source: Alzheimers Res Ther. 2019 Jun 29;11:57. doi: 10.1186/s13195-019-0512-1 (PMC6599313; doi:10.1186/s13195-019-0512-1)
Supplement: Supplementary file 4 — Clinical progression between every pair of F+ and F− groups. (DOCX 265 kb) [file 13195_2019_512_MOESM4_ESM.docx]

**
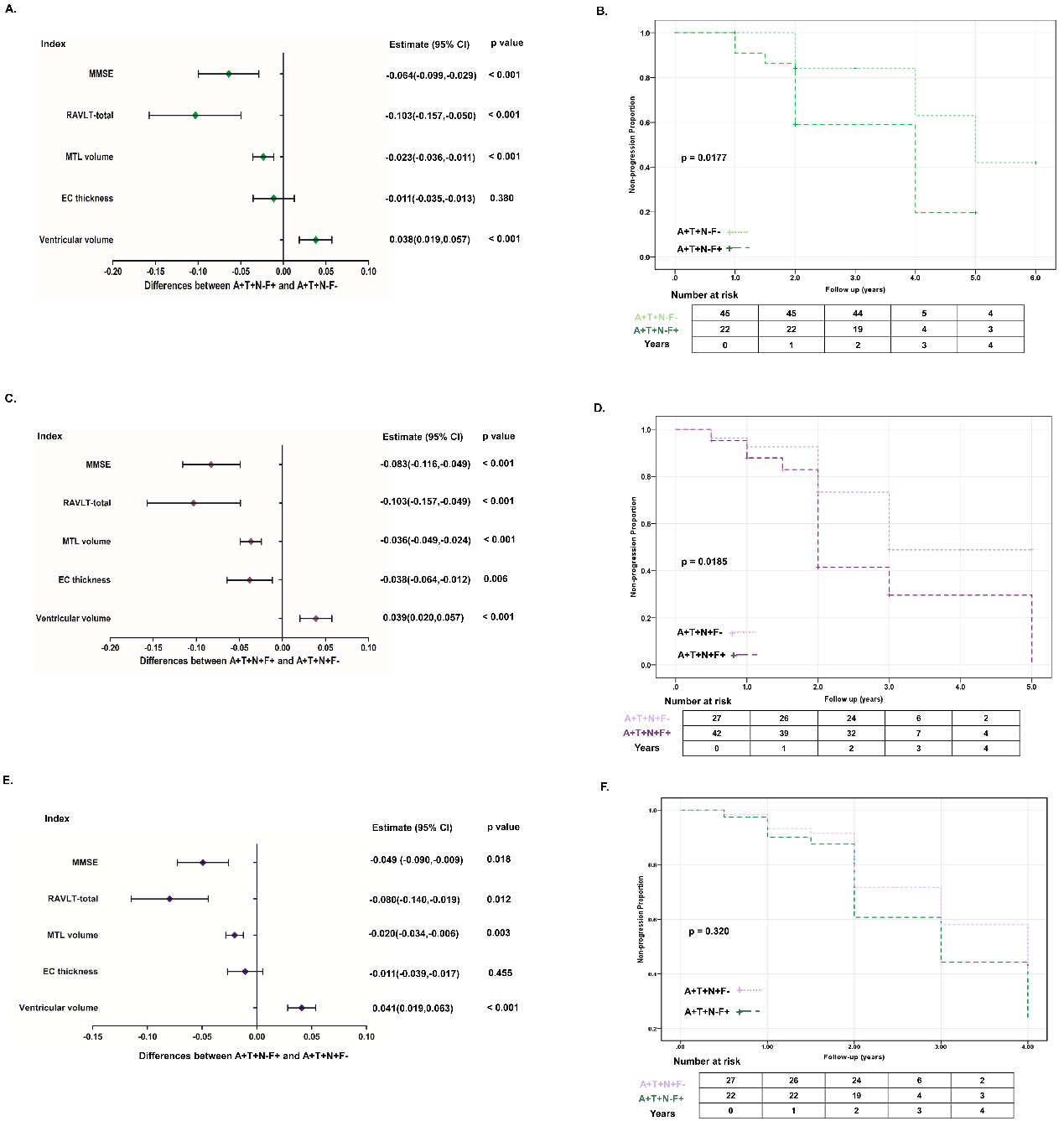
**

**Additional file 4 Clinical progression between every pair of F+ and F- groups**

Panels A, C, E showed the comparisons of longitudinal changes in cognitive performances and brain structure with A+T+N-F- vs A+T+N-F+ revealed in panel A, A+T+N+F- vs A+T+N+F+ in panel C, and A+T+N+F- vs A+T+N-F+ in panel E. Differences between every pair of F+ and F- subgroups were demonstrated by estimates with 95% CIs, and p values. Analyses of cognitive decline were adjusted for age, gender, APOE ε4 and years of education. Analyses of brain atrophy were adjusted for age, gender, APOE ε4 and total intracranial volume.

Panels B, D, F demonstrated the Kaplan-Meier curves showing cumulative probability of MCI-to-AD dementia progression, which were arranged in accordance with the order mentioned above. The small crosses are censored data, and the number of subjects at risk is noted at the bottom of the plot. The unadjusted p values of log-rank test were depicted in the lower left.

Abbreviations: MMSE: Mini-Mental State Examination, RAVLT: Rey Auditory Verbal Learning Test, MTL: middle temporal volume, EC: entorhinal cortex, MCI: mild cognitive impairment, AD: Alzheimer’s disease.
