# Supplementary material for: FDG-PET as an independent biomarker for Alzheimer’s biological diagnosis: a longitudinal study
Source: Alzheimers Res Ther. 2019 Jun 29;11:57. doi: 10.1186/s13195-019-0512-1 (PMC6599313; doi:10.1186/s13195-019-0512-1)
Supplement: Supplementary file 5 — Comparison of clinical progression between CSF T-tau (+) vs HVa (+) vs FDG-PET (+). (DOCX 1321 kb) [file 13195_2019_512_MOESM5_ESM.docx]

**
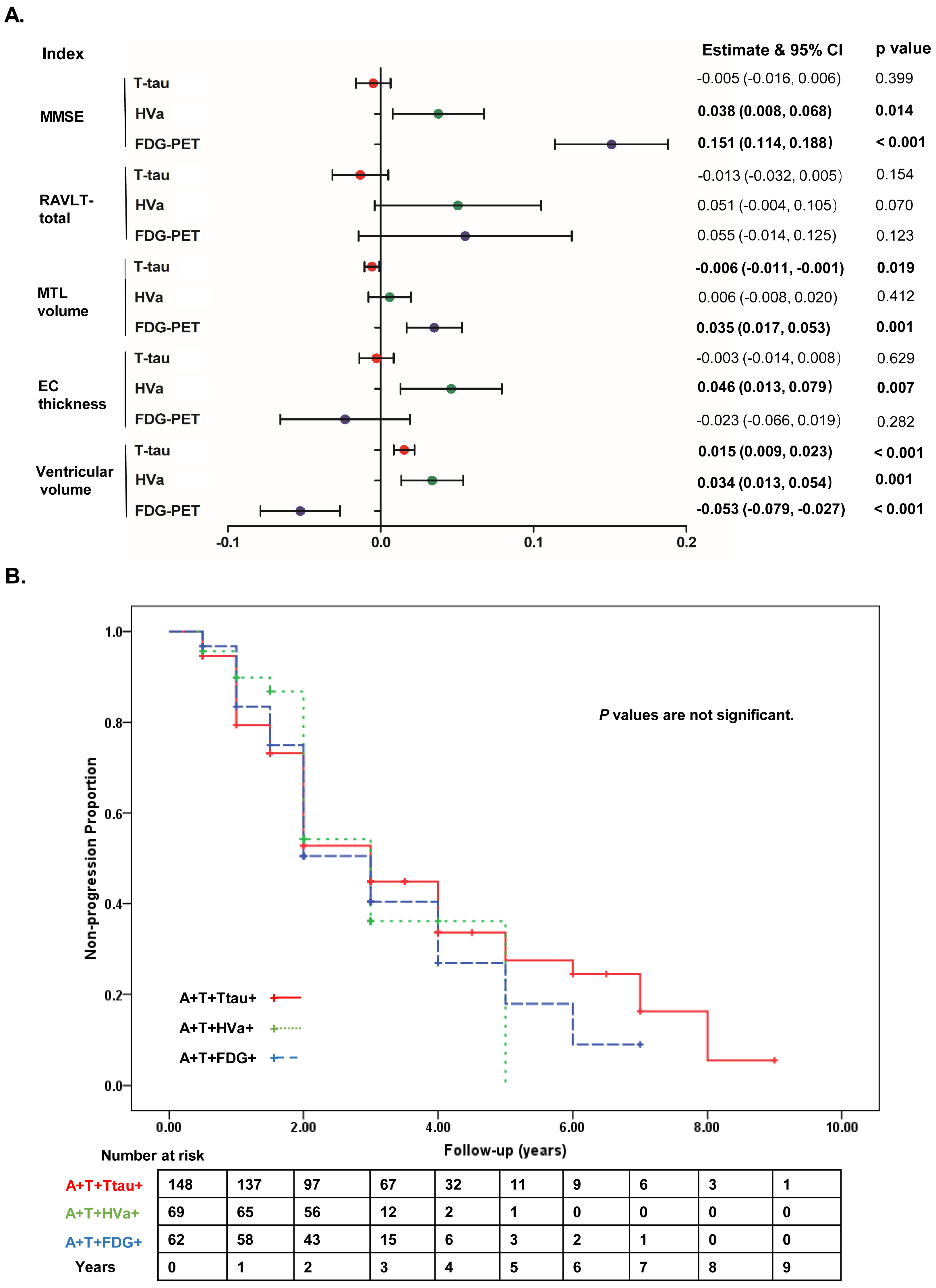
**

**Additional file 5 Comparison of clinical progression between CSF T-tau (+) vs HVa (+) vs FDG-PET (+)**

Panel A showed the longitudinal correlations between the three “N” biomarkers with cognitive decline and brain atrophy. Analyses of cognitive decline were adjusted for age, gender, *APOE* ε4 and years of education. Analyses of brain atrophy were adjusted for age, gender, *APOE* ε4 and total intracranial volume.

Panel B showed the Kaplan-Meier curves of MCI-to-AD dementia conversion in the A+T+Ttau+, A+T+HVa+, and A+T+FDG+ groups. The small crosses are censored data, and the number of subjects at risk is noted at the bottom of the plot.

Abbreviations: MMSE: Mini-Mental State Examination, RAVLT: Rey Auditory Verbal Learning Test, MTL, middle temporal volume, EC, entorhinal cortex, T-tau, total tau, HVa, adjusted hippocampal volume, FDG-PET: 18F-fluorodeoxyglucose-positron emission tomography, MCI: mild cognitive impairment, AD: Alzheimer’s disease.
